# Effect of the Addition of Agribusiness and Industrial Wastes as a Partial Substitution of Portland Cement for the Carbonation of Mortars

**DOI:** 10.3390/ma14237276

**Published:** 2021-11-28

**Authors:** Wilfrido Martinez-Molina, Hugo L. Chavez-Garcia, Tezozomoc Perez-Lopez, Elia M. Alonso-Guzman, Mauricio Arreola-Sanchez, Marco A. Navarrete-Seras, Jorge A. Borrego-Perez, Adria Sanchez-Calvillo, Jose A. Guzman-Torres, Jose T. Perez-Quiroz

**Affiliations:** 1 Faculty of Civil Engineering, Universidad Michoacana San Nicolas de Hidalgo, Morelia 58070, Mexico; elia.alonso@umich.mx (E.M.A.-G.); mauricio.arreola@umich.mx (M.A.-S.); mnavarrete@umich.mx (M.A.N.-S.); jorge.borrego@umich.mx (J.A.B.-P.); jaguzman@umich.mx (J.A.G.-T.); 2 Centro de Investigación en Corrosión, Universidad Autónoma de Campeche, Campeche 24070, Mexico; tezperez@uacam.mx; 3 Faculty of Architecture, Universidad Michoacana San Nicolas de Hidalgo, Morelia 58070, Mexico; adria.sanchez@umich.mx; 4 Coordinación de Ingeniería Vehicular e Integridad Estructural, Mexican Institute of Transportation (IMT), Queretaro 76703, Mexico; jtperez@imt.mx

**Keywords:** durability, residues, carbon dioxide, pollution, porosity, pozzolanic activity

## Abstract

The present research work shows the effect on the carbonation of Portland cement-based mortars (PC) with the addition of green materials, specifically residues from two groups: agricultural and industrial wastes, and minerals and fibres. These materials have the purpose of helping with the waste disposal, recycling, and improving the durability of concrete structures. The specimens used for the research were elaborated with CPC 30R RS, according to the Mexican standard NMX-C-414, which is equivalent to the international ASTM C150. The aggregates were taken from the rivers Lerma and Huajumbaro, in the State of Michoacan, Mexico, and the water/cement relation was 1:1 in weight. The carbonation analyses were performed with cylinder specimens in an accelerated carbonation test chamber with conditions of 65 +/− 5% of humidity and 25 +/− 2 °C temperature. The results showed that depending on the PC substitutions, the carbonation front advance of the specimens can increase or decrease. It is highlighted that the charcoal ashes, blast-furnace slags, and natural perlite helped to reduce the carbonation advance compared to the control samples, consequently, they contributed to the durability of concrete structures. Conversely, the sugarcane bagasse ash, brick manufacturing ash, bottom ash, coal, expanded perlite, metakaolin, and opuntia ficus-indica dehydrated fibres additions increased the velocity of carbonation front, helping with the sequestration of greenhouse gases, such as CO_2_, and reducing environmental pollution.

## 1. Introduction

In the world, the most employed construction material is the hydraulic concrete Portland cement (PC). Due to its great mechanical performance, low cost, durability and versatility, PC is used for all types of structures and construction purposes. In the plastic state, it can take any geometric form or design, adapting to the formworks or shoring, later curing and acquiring the desired mechanical resistance.

Concrete consists of coarse aggregate (gravel), fine aggregate (sand), water, PC, and eventually different admixtures. After the curing process, concrete transforms into an artificial rock material. If it is reinforced with steel cores, it is named reinforced concrete; this composite material combines the major uniaxial compression resistance of concrete with the elasticity, resiliency and ductility of the steel reinforcements, allowing to build structures with great durability properties.

Nevertheless, the PC materials, such as concrete and construction mortars, generate a big environmental impact, especially during the producing of the clinker. For each 1000 kg of clinker produced, 600 to 800 kg of CO_2_ are emitted, making the cement industry one of the most pollutant over the world, contributing to the 8% of the total CO_2_ worldwide emissions [1]. The extraction and processing of the prime materials for the elaboration of PC also contributes to this pollution issue. In addition, the petrous aggregates are non-renewable materials, and the exploitation of the quarries causes a negative environmental impact. Many studies have warned from the importance of diminish the effects of the PC industry on the natural environment [2,3].

Besides all this situation, the durability and preservation of the concrete is severely affected by the adverse atmospheric conditions. The durability is the capacity of a construction material, element or structure to resists the physical, chemical, biological, environmental and global warming actions during a determined and designed period of time, while preserving its original form, mechanical properties and service conditions [4].

An aggressive ambient, which contains chloride ions, can be found in constructions such as the docks or bridges built in coastal regions, due to the high salinity of the water and the sea breeze [5]. In addition, the structures built over soils with greater sulphate content can be prone to suffer damages which affect their durability [6]. The cities or industrial zones where the CO_2_ content in the atmosphere is elevated suffer from similar problems. When the gases penetrate the concrete through the pores of the material, the CO_2_ chemically react with the calcium hydroxide and the calcium silicates, which are products of the reaction of the PC with the water during the hydration process of concrete, forming calcium carbonate. This chemical reaction reduces the pH of the material, which contributes to a faster degradation by corrosion of the reinforced steel, compromising the structural integrity of the constructions [4]. Other environmental factors such as the CO_X_ are precursors of the carbonation of the reinforced steel of concrete structures [3].

This phenomenon may affect the stability of the structural reinforcements and produces the oxidation and depassivation processes [7,8], starting major damages in civil and industrial structures. The inclusion and research of additives and substitutions to suppress the carbonation allows us to increase the durability of reinforced concrete constructions and helps to diminish the exploitation of the petrous aggregate quarries. The simplest way to achieve this is to maintain a low water/cement ratio and to comply with good construction practices and regulations with correct quality management.

The prevention of corrosion can be achieved mainly during the design phase using high quality concrete with the adequate covering; this approach has been standardized in the Eurocode 2 and the standard EN 206 [7,8,9]. It is important, because the majority of the corrosion damages are caused by poor design and execution of the concrete (positioning, compaction and curing). Regarding the quality of the concrete, the benefits from and low water-binder ratio and its permeability are well known [7,9,10].

Nevertheless, it is possible to benefit from the carbonation process of the materials and learn from the previous research which has obtained encouraging results. Chen and Gao (2019), for example, found that the water loss of the PC pastes from 30% to 40% is optimal for the CO_2_ absorption [11]. The combination of a correct precuring and carbonation period can also increase the uniaxial compressive strength of the PC mortars effectively, especially at early ages; the PC pastes can improve their mechanical resistance and microstructure with this method [11]. Other papers point with thermogravimetric analysis that the carbonation process can delay the hydration of the cement pastes and with higher CO_2_ concentrations the crystallinity degree of the carbonates increases. Conversely, with energy-dispersive X-ray spectroscopy (EDS), it is possible to determine if an excessive carbonation of the material can cause a decalcification of the calcium silicate hydrate (C-S-H), which is the core of the resistance and durability of the PC mixtures.

Different works have studied and documented the mechanical properties and the porosity of PC pastes with high resistance to sulphates (HS SR PC) subdued to curing by carbonation at early ages. One study case submitted two pastes to different carbonation ages (1 and 24 h) [12]. It was found that the sample with 1 h improved its mechanical properties, while the other sample reduced them in comparison with the reference samples. Besides, it was found that the increase of the carbonation time from 1 to 24 h enhanced substantially the absorption properties. Notwithstanding, the higher mechanical resistance of the one-hour sample also entailed a higher content of absorbed water compared with the reference test.

Currently, the employment of PC presents some challenges to diminish the carbon footprint. Some of the approaches to achieve it include the incorporation of materials which act as partial replacements of PC [13], to reinforce circular economy, improve the physical, mechanical and chemical properties, reduce the relation water/cement of concrete, and the retardation of the carbonation [14,15]. Several research works have explored these strategies with encouraging results and publications relying on materials with pozzolanic activity [16,17,18,19,20,21,22]. The origin of the pozzolanic activity materials lies in the locality of Pozzuoli, Italy, where the volcanic activity of the region produced these materials which present great cementitious properties only after reacting with calcium hydroxide. 

Another strategy is the utilization of waste materials such as glass [23], PET bottles or containers [24], ceramic tiles and coverings, ceramic sanitary products [25], ceramic clay bricks [26], tyres and rubber [27], concrete residues [28], agricultural wastes [29,30], metallurgy slags [31,32,33], industrial wastes [34,35,36], or coconut husks [37]. All these additions proportionate a positive environmental impact due to the reutilization of materials and products which had completed their service life. These waste materials are a viable alternative for construction purposes, as many investigations have proven improvements in the mechanical resistance, durability and elasticity while reducing the exploitation economic costs [38,39,40,41,42,43,44]. Conversely, many of the substitutions may reduce the workability of the concrete casting and diminish the tensile strength [45,46].

In addition, the diverse mineral additions have been researched with binary combinations of PC (silica fume, metakaolin, fly ash, among others). They have been studied by means of the monitoring and analysis of thermodynamic and kinetic parameters, and the evaluation of the corrosion process in reinforced concrete specimens subdued to prolonged chloride attack (44 weekly moistening cycles with a NaCl solution and air-dried, the equivalent of 308 days’ chloride attack). The electrochemical techniques: Ecorr, Rp and EIS (Electrochemical Impedance Spectroscopy), in combination with the electrical resistivity, have helped to evaluate the protection capabilities of the studied concretes, regarding the strong environmental attacks.

The mineral additions have produced meaningful gains in the resistivity of the concretes, mainly due to the physical alterations of the pore structure of PC pastes. The additives also cause an enhancement of the interphase paste-aggregate and the conductivity of the porous solution.

The employed mineral additions have significantly increased the resistivity of concretes, due to the physical alterations in the porous structures of the PC pastes, the improvement of the paste-aggregate interphase, and the conductivity changes of the porous solution [47].

The carbonation process absorbs the CO_2_ in the atmosphere, compensating partially the polluting emissions generated during the cement production [48,49]. Jacobsen and Jahren [50] estimated that the 16% of the CO_2_ emissions of PC production are reabsorbed during the service life of the material and this carbonation process. Recent works and additions, such as glass powder wastes from organic light-emitting diodes (OLED), have demonstrated a greater CO_2_ absorption while carbonation process [51]. 

Recently, the study of using waste materials to enhance the carbonation resistance in mortars has attracted attention in order to improve the properties of mortars [52,53,54,55,56,57]. Recent studies have analysed the acceleration of carbonation of two types of lime mortars (standard sand and extra ceramic dust) [58]; the mortar with extra ceramic dust showed advanced calcite precipitation and early improvement in various properties. Dvender Sherma and Shweta Goyal used cement kiln dust as partial replacement of cement and an accelerated carbonation curing process to improve the compressive strength, and they studied the porosity and pH of the mortar mix [59]; the results showed an increase of 20% in strength and a reduction of the porosity. Ioannis Rigopoulos et al. studied the carbonation of air lime mortars modified with quarry waste [60], rich in Ca, Mg and Fe silicate minerals. It has been demonstrated how the incorporation of quarry wastes increases the compressive strength and density the microstructure of the mortar. Shan Liu et al. analysed the adding of different additives (calcined hydrotalcite, calcium silicate, gypsum and silica fume) [60], on alkali-activated mortar to enhance the carbonation resistance; the results showed that the incorporation of calcined hydrotalcite improved the carbonation resistance and compressive strength.

## 2. Materials and Methods

The study aim is to analyse the carbonation behaviour of mortars by adding different waste materials and configurations with cementitious and/or pozzolanic properties as substitutions of PC. It was determined if such additions can limit or enhance the carbonation of the mixtures when hardened.

### 2.1. Sampling Materials and Selection

A regional study was made in the state of Michoacan, Mexico, to find the most abundant and suitable solid wastes and the market necessities and opportunities. The residues were characterized in the laboratory of the Faculty of Civil Engineering of the university “Universidad Michoacana San Nicolas de Hidalgo” to determine their cementitious capacity and their feasibility as construction materials. The additions were studied as partial substitution of PC with different percentages; the effects on the mechanical properties of the mortars were monitored.

The PC substitutions were considered among two main groups: wastes from agricultural and industrial processes, and mineral origin materials and fibres. All the additions were compared with control samples elaborated with cement CPC 30 R RS according to the Mexican code NMX-C-414 [61], directly related with cement Type 4 of the international standard ASTM C-150 [62]. Table 1 shows the list of materials analysed for mortar mixtures in Mexico. The table displays the origin of each addition and the percentage added as PC substitution (%).

Sands from two rivers located in the Mexican state of Michoacan were used for the preparation of the different mixtures in order to compare the influence that each type of sand has on the different mortars studied. C1 are core mortars made with sand from the Lerma river and C2 are core mortars made with sand from the Huajumbaro river. The mortars made with sand from the Lerma river are: BMA (Brick manufacturing ash), BA (Bottom ash), C (Coal), OF (Opuntia ficus-indica dehydrated fibres). Conversely, the mortars made with sand from the Huajumbaro river are: SBA (Sugarcane bagasse ash), CA (Charcoal ashes), BMA (Brick manufacturing ash), EP (Expanded perlite), NP (Natural perlite), MK (Metakaolin).

### 2.2. Elaboration of the Specimens

The mortars were designed with a 1:2.5 cement/aggregate proportion and a 1:1 water/cement ratio in weight. The purpose was to achieve good workability and fluidity (including the specimens with incorporation of the substitutions). The studied materials were added as a partial replacement of the PC.

The mortars were elaborated under lab conditions, dosed by weight, mechanically mixed, with safe water, and with the dosages and substitutions shown in Table 1. The specimens designed for the accelerated carbonation analysis were cylinders of 5 cm diameter and 10 cm height. All the specimens were subdued to curing process by immersion after the uncasing, according to the standard ASTM C31 [63].

### 2.3. Carbonation Analysis

**Figure 1:**
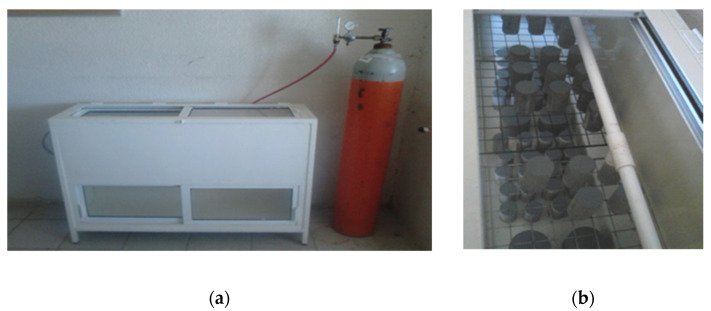
(**a**) Accelerated carbonation chamber, ACC. (**b**) Samples distribution in ACC.

**Figure 2:**
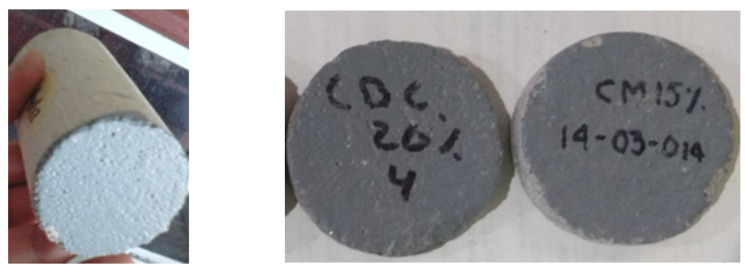
Vinyl paint applied in cross sections of cylinders.

**Figure 3:**
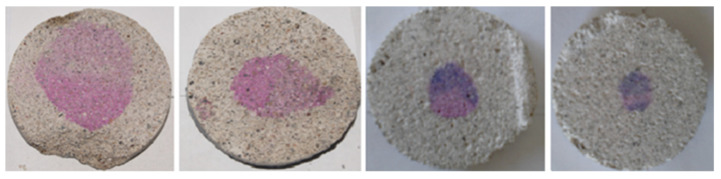
Carbonation monitoring by using phenolphthalein and thymolphthaleine.

After that, they were exposed in series of four elements to the carbonation chamber with a relative humidity of 65 +/− 5%, temperature of 25 +/− 2 °C and 3% of CO_2_, (See Figure 1). Cylinders were covered with vinyl paint in their both cross sections, as is shown in Figure 2, to prevent the carbonation advance through the longitudinal axis. After being subjected to the accelerated carbonation, each series of cylinders was cut into 5 mm slices and sprinkled in the exposed side with the indicators (phenolphthalein and thymolphthalein-based), to measure the carbonation front, as shown in Figure 3. The measurements were taken at 30, 60, 90, 120, and 180 days.

### 2.4. Compressive Strength

**Figure 4:**
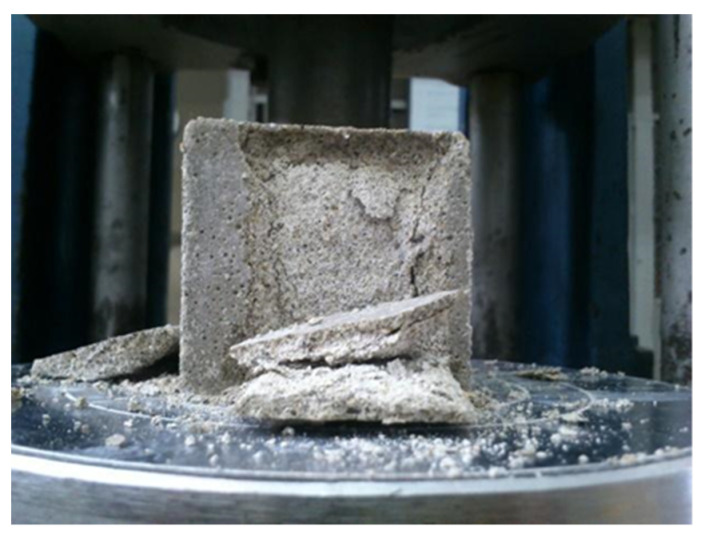
Illustration of the compressive strength test.

To complement the study of the effects on the properties of the researched materials, the uniaxial compressive strength was applied to the specimens, as is shown in Figure 4. This test, as well as the elaboration of the samples, were performed following the standards ASTM C109/109M [64].

### 2.5. Electrical Resistivity Test

**Figure 5:**
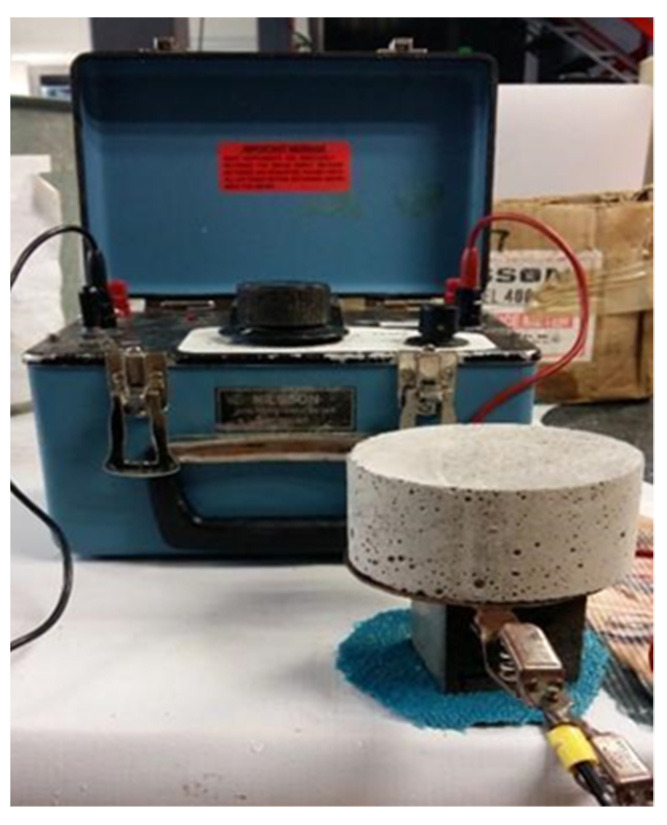
Illustration of the electrical resistivity test and equipment.

In addition to the compressive resistance, the electrical resistivity test was applied to the samples, as can be seen in Figure 5. The test was performed with the same specimens used in the mechanical analyses following the standards ASTM C1876 and the DURAR manual [44,65].

## 3. Results

### 3.1. Characterization of the Aggregates

Table 2 presents the mechanical properties of the sand aggregates employed in the concrete mixtures. Regarding the absorption percentage and the sand equivalent value we find significant differences; nevertheless, the rest of the properties remain equivalent.

In the characterization of the sands of the Huajumbaro and Lerma rivers, it was found that both materials are composed of silica and have similar physical properties. Table 2 shows significant differences in the absorption percentage and the clay content of both samples, which probably has a repercussion on the carbonation process and evolution and the k constant, later presented in this research work. In addition, their mechanical behaviour in compression is very similar, where the mortars made with both sands reached the same strength (13.3 MPa).

### 3.2. Characterization of the Additions

**Figure 6:**
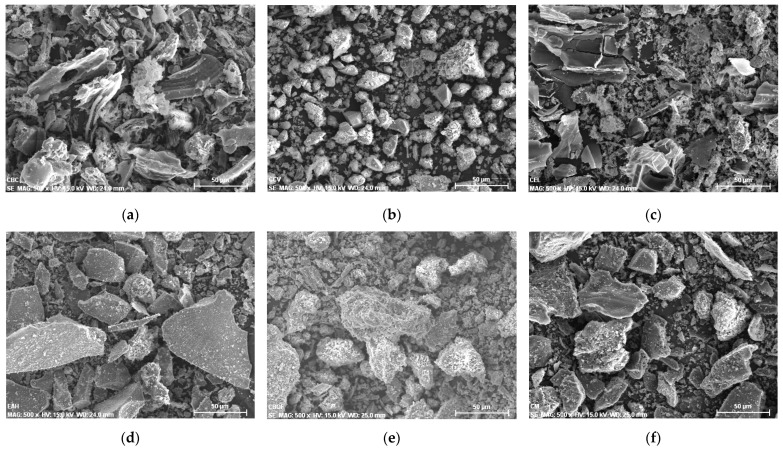
SEM images of the substitution materials. (**a**) SBA; (**b**) CA; (**c**) BMA; (**d**) BFA; (**e**) BA; (**f**) C.

**Figure 7:**
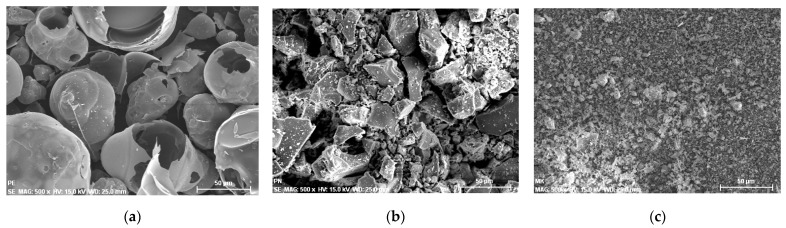

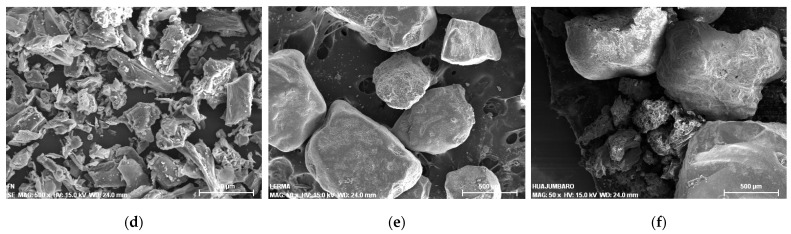
SEM microscopy images of the substitution materials. (**a**) PE; (**b**) NP; (**c**) MK; (**d**) OF; (**e**) C1; (**f**) C2.

The PC substitution materials studied in this research were analysed with SEM microscopy to observe the granulometry and particle size of each one of them. In Figure 6a–f and Figure 7a–f several morphologies and particle shapes are displayed, with well-defined particles with diameter measurements between 40 and 60 µm. The BMA and SBA samples show a composition of morphologies with great surface area, while conversely, the CA sample presents more scattered conglomerates, with smaller size particles. This variety of size, morphology and surface area provides important information to later analyse the mixtures with PC.

**Figure 8:**
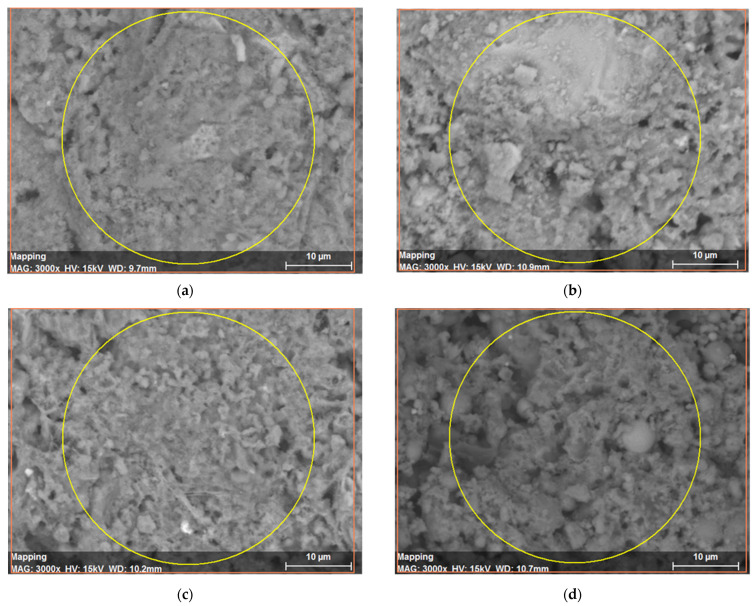
SEM images of the mortar samples with different substitution materials. (**a**) BMA 5%; (**b**) SBA 5%; (**c**) CA 5%; (**d**) BFS 15%.

The samples were characterized by scanning electron microscopy—energy dispersive X-ray spectroscopy (SEM-EDS). The images in Figure 8a–c show the morphology of the mortars with a considerable surface area and conglomerate compounds of petrous materials and cement. Most of the samples present porous surfaces and voids filled with air during the solidification. Overall, the samples show good homogenization and coupling regarding the proposed design and additions.

In Table 3, the results of the EDS mapping and the elemental composition of the substitution samples are shown. They are expressed in percentage by total weight.

A variety in the composition of the substitutions can be noted. SiO_2_, Al_2_O_3_ and Fe_2_O_3_ are chemical compounds with high pozzolanic activity, and their presence in all the addition materials is significant. Table 4 presents the pozzolanic activity value of the materials, being MK (94.969), EP (88.186), NP (86.791) and SBA (69.474) the ones with major values. This composition of the additions allows subsequent reactions to the hydration of the cement compounds, providing improvements in the mechanical resistance and a reduction of the porosity. Nevertheless, these secondary reactions are conducted with the alkalises generated during the cement hydration, with the disadvantage of reducing the pH and the concentration of alkaline species which confer high pH values to the fresh concrete.

As expected, the mortars with additions reduced their porosity and increased their uniaxial compression strength. Nevertheless, some authors mention that, in few cases, the results can be opposed [13,14]. Besides, the referred secondary reactions of the pozzolanic compounds consume the alkalis existing in the pores dissolution and can generate a decrease of the pH. Regarding this alkalis generation, BFS and BA present higher contents of MgO and CaO; and NP has 3.216% Na_2_O and 4.285% K_2_O. The presence of these compounds underwater could be a great source of alkalis generation, which would keep the pH over 12, even after the pozzolanic reactions.

In respect of the aggregates, their composition with great siliceous content makes them susceptible to experiment alkali-silica reactions, which is one of the main causes of severe damages in concrete structures. However, the silica of these materials is present in quartzes; consequently, they do not react, but otherwise, they neither achieve the pozzolanic activity due to the crystallinity properties. Then, the aggregates work as inert materials.

### 3.3. Carbonation Analysis

For a proper analysis of the figures and tables presented in this section, it is important to make clear that each substitution sample is compared with the control samples.

**Figure 9:**
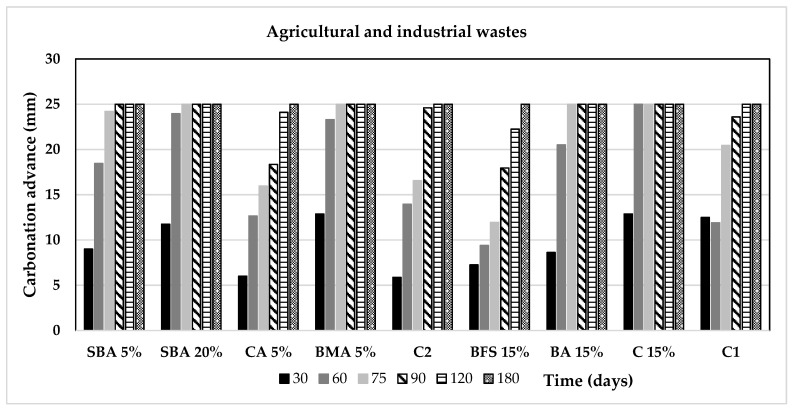
Advance of the carbonation front as a function of the time for the agricultural and industrial wastes group of additions.

**Figure 10:**
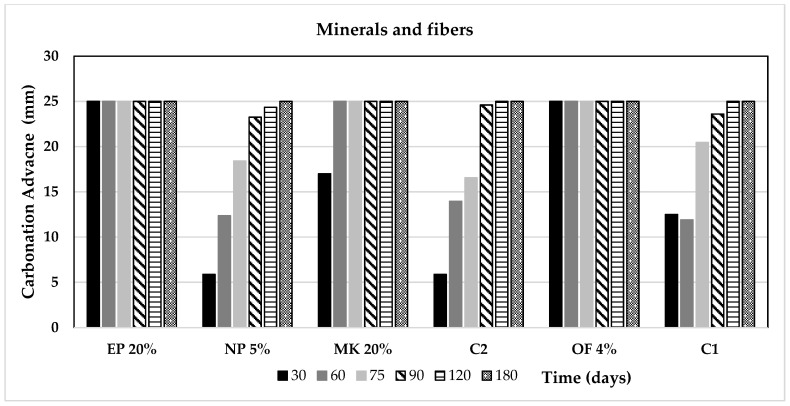
Advance of the carbonation front as a function of the time for the minerals and fibres group of additions.

Figure 9 and Figure 10 display the advance of the carbonation front of the mortar samples of this research. It can be noted that all the samples met 25 mm of carbonation front in the maximum period of 180, although some of them achieved it earlier; this measurement is important since 25 mm is the most common depth of the reinforced steel in Mexican concrete structures.

**Figure 11:**
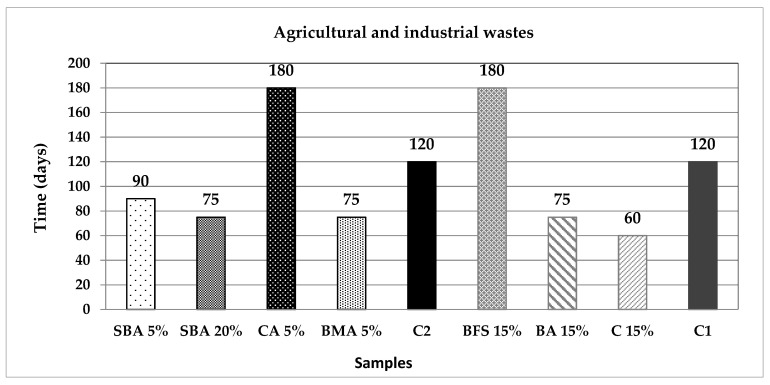
Critical time to reach out 25 mm of carbonation front, for the agricultural and industrial wastes group of additions.

**Figure 12:**
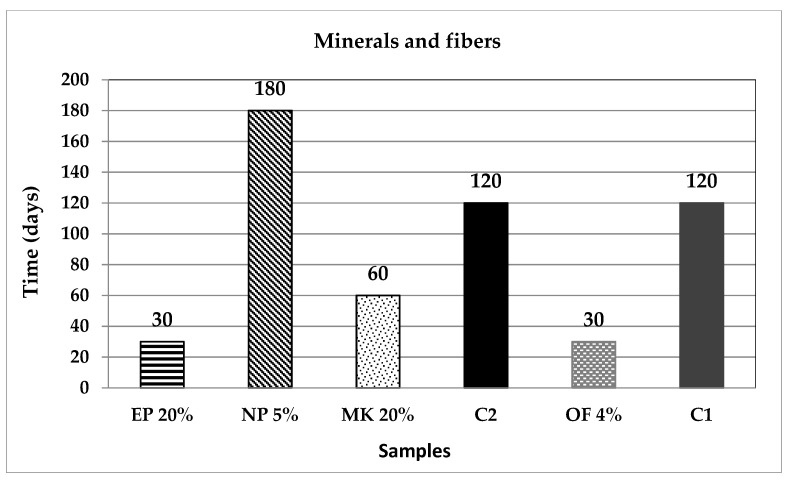
Critical time to reach out 25 mm of carbonation front, for the minerals and fibres group of additions.

Figure 11 and Figure 12 present the required time to reach out the mentioned 25 mm of carbonation front. The EP and OF samples achieved the mark within the initial period of 30 days; the MK and C samples reached it at 60 days; SBA5, BMA, and BA substitutions lasted 75 days to carbonate; at 90 days the SBA20 met the mark; C1 and C2, the control samples needed 120 days; and at last, BFS, CA and NP reached the total carbonation at 180 days. Taking this parameter as an indicator of the concrete quality, the carbonation sequence indicates which additions are the better to increase the durability of structures. Conversely, the samples that reached carbonation in lesser time periods could be useful for carbon dioxide reduction and sequestration, helping to absorb these greenhouse gas emissions.

Another way to quantify and correlate the carbonation advance in concrete structures is the following equation:

e = kt^1/2^ (1)

where:

e = carbonation front depth, in mm;

k = proportionality constant of the carbonation process;

t = exposure time, in years.

**Figure 13:**
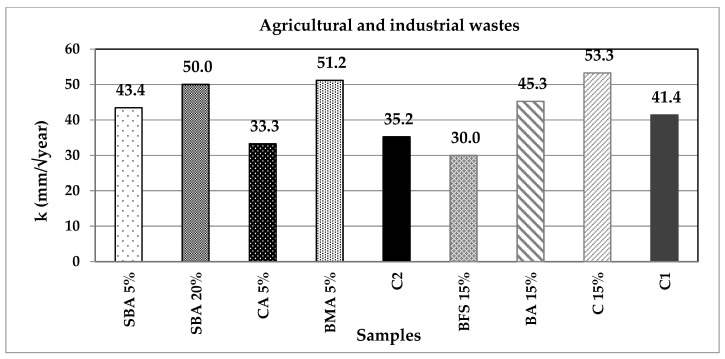
Constant k for the agricultural and industrial wastes group of additions.

**Figure 14:**
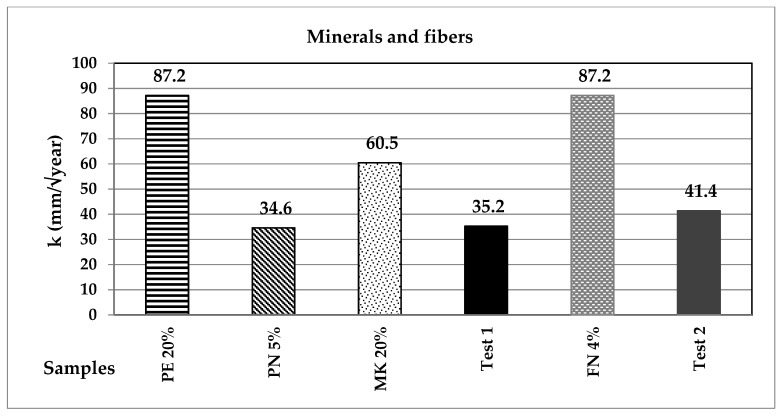
Constant k for the minerals and fibres group of additions.

Figure 13 and Figure 14 display the value of constant k (in mm/year^2^) for all the specimens. According to the equation, it can be noted that the higher k values correspond to the mortars with faster carbonation times. CA, BFS and NP reduced the carbonation velocity in comparison with the control samples, C1 and C2; therefore, these substitutions are suitable to increase the durability of reinforced concrete structures. It can also be noted that there is not a definite trend regarding the carbonation rapidity depending on the incorporated additions.

According to the manual of the DURAR network [44,74], in natural conditions, the carbonation coefficients above 6 mm/year^1/2^ are representative of bad quality concretes, and below m mm/year^1/2^ are representative of high quality concretes. In the present study case, the CO_2_ experimental concentration was 3%, which cannot be compared with the natural exposure of concrete structures.

In the case of the samples SBA, BMA, BA, C, EP, MK and OF, it is apparent that the incorporation of these substitutes furthers the carbonation of the mortar mixtures to a greater or lesser extent. The EP and OF samples, which reached out the 25 mm carbonation front in only 30 days (three times faster), standing out from the rest; MK and C also achieve the carbonation two times faster than the control samples. This quick carbonation may be due to the high porosity of the mixtures and the low reactivity of the pozzolanic compounds with the alkalis existing after the hydration of the PC.

**Figure 15:**
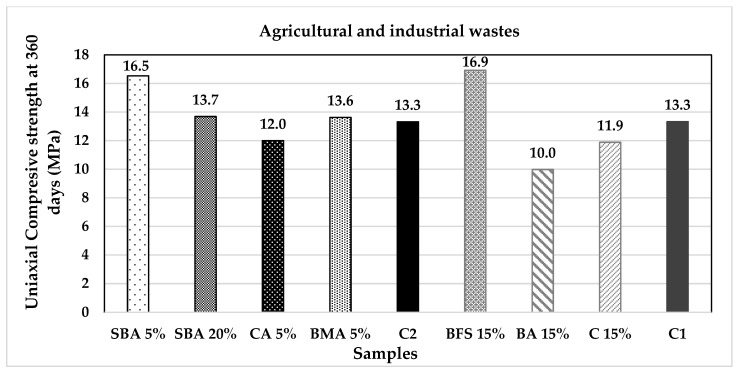
Uniaxial compressive strength of agricultural and industrial wastes group of additions.

**Figure 16:**
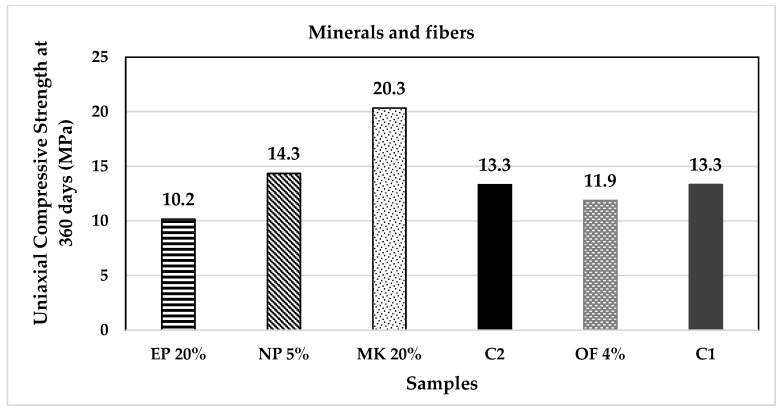
Uniaxial compressive strength of minerals and fibres group of additions.

Figure 15 and Figure 16 present the uniaxial compressive strength values of the analysed specimens; it is notable that the mixtures with higher k values and lower carbonation times also present lower mechanical resistances compared to the control samples. Two samples stand out from the rest: SBA and MK; they present higher mechanical properties while at the same time promote carbonation; in the case of SBA, it can be observed how the greater is the substitution percentage, the higher is the carbonation velocity and the lower is the mechanical resistance.

Other substitution material with an unusual behaviour was CA, showing lower mechanical values than control samples but with a low carbonation velocity, being one of the three materials that achieve in reducing it. CA may act as a filler and not as a pozzolan, allowing to limit the interconnection of the cementitious matrix pores and increase its tortuosity without actually densifying the matrix enough to bring more mechanical resistance.

**Figure 17:**
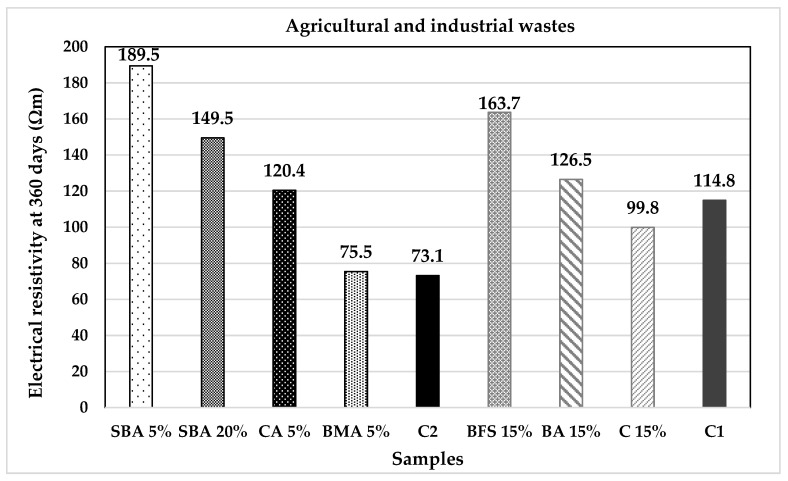
Electrical resistivity of the agricultural and industrial wastes group of additions.

**Figure 18:**
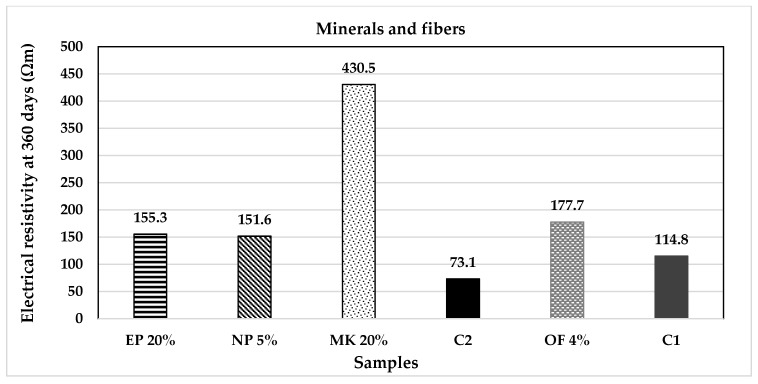
Electrical resistivity of the mineral and fibres group of additions.

At last, the results of the electrical resistivity test are shown in Figure 17 and Figure 18. We can observe that all the substitutions proposed present better behaviours than the control samples. Similar than Figure 15 and Figure 16, SBA and MK have higher values of electrical resistivity and uniaxial compressive strength while they favour the faster carbonation. It is known that SBA contain amorphous SiO_2_, which acts as a puzzolana reacting with Ca(OH)_2_ produced during the hydration process of cement and water, acting as a retarding reaction, resulting into the generation of a saturated zone of calcium silicate hydrate (CSH) gel, selling porous and increasing compression resistance. This CSH gel reduces the amount of calcium hydroxide Ca(OH)_2_ and consequently the pH of the cementitious paste [75]. Metakaolin (MK), Al_2_Si_2_O_7_, is a highly amorphous dehydration product of kaolinite, Al_2_(OH)_4_Si_2_O_5_; it contains silica and alumina in an active form which will react with CH. For MK, similar pozzolanic reaction occurs through its interaction with the calcium hydroxide present in the cement paste, forming hydrated calcium silicates (C-S-H) and aluminates (C_2_ASH_8_, C_4_AH_13_ and C_3_AH_6_) [76]. Both material samples were exposed in the accelerated carbonation chamber under experimental temperature, relative humidity and CO_2_ concentration. This favoured the Ca(OH)_2_ reactions, reducing their pH and increasing their mechanical resistance. This demonstrates that both materials have a high potential as CO_2_ environmental recruiters without compromising the mechanical properties of the concrete mixtures.

The results can be correlated with the origin of the substitution materials. The materials with organic and mineral origin, as well as the agricultural and industrial wastes, can increase or decrease the carbonation process and with that modify the quality of the concrete in terms of durability. Only NP, CA and BFS improved the carbonation resistance in comparison with the control samples; the rest of additions presented higher k values, not being recommendable for high quality structures which require great durability.

According to the elemental composition of samples and the pozzolanic activity, SBA, MK and EP react with the alkalis of the cement pastes, while Ca(OH)_2_, NaOH and KOH reduce the capability to resist the carbonation advance. In the case of NP, the alkalis quantity, 3.216 Na_2_O and 4.285 K_2_O, have the capacity to generate NaOH and KOH when they come into contact with water and then provide chemical reactions to the concrete to reduce the carbonation velocity. Regarding the pozzolanic activity of CA (51.44) and BFS (47.35), which are the other two materials that improved the carbonation resistance, they do not consume great amounts of alkalis; furthermore, they present higher concentrations of NaOH (2.108 and 10.107) and KOH (18.759 and 37.551), respectively.

It is important to mention that the criteria for the selection of substitutions and mixtures is not subdued to the carbonation velocity. A designed mixture could achieve great mechanical properties but suffer carbonation easily; in that case, it is recommended to apply superficial refurbishments and protect the structure.

**Figure 19:**
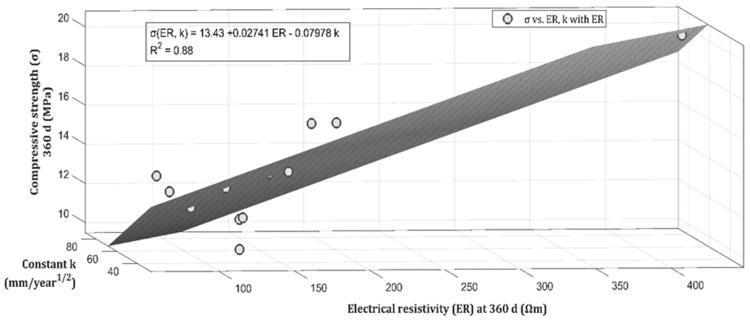
Multi-variable linear regression estimating the compressive strength d.

The numerical analysis is an important task employed in this kind of studies in order to establish or find some relation between the features analysed. The present study establishes a multivariate analysis employing the method of least squares to estimate the compressive strength of the samples, as it is shown in Figure 19.

The figure displays the performance of the linear regression model depicted in a plane that fits all the data analysed. The electrical resistivity (ER) and the constant k are the two predictive variables used to build the multivariate regression model. This model yields a linear equation which is described in Equation (2).

σ (ER, k) = 13.43 + 0.02741ER − 0.07978k(2)

One of the most common metrics for evaluating the accuracy of a regression model is the determination coefficient R^2^. This coefficient provides the proportion of the entire variance involved by the regression task and is related with the accuracy achieved by the model [77]. The coefficient of determination can be used to evaluate the goodness-of-fit of the model and it always varies between 0 and 1. When R^2^ is equal to 1, it means that there is a perfect correlation and that the sample data lie exactly on the regression line [78]. For this case the coefficient value is equal to 0.88, which means that the plane captures 88% of the information correctly. Furthermore, it is crucial to establish that the compressive strength in mortars is a value that is possible to estimate with acceptable accuracy (88%) just using the electrical resistivity value and a constant k with a simple linear multivariate regression. Nevertheless, the equation can be improved by exploring a superior order regression, a task which will be completed in further research projects.

## 4. Conclusions

From the eleven total materials studied in this research as partial substitutions of PC for concrete mixtures; three of them showed to reduce the carbonation velocity (CA, BFS, and NP) presenting lower k carbonation coefficients than the control samples without additions. The other eight substitution materials presented higher carbonation values; however, the whole of the specimens partially substituted the PC, allowing to decrease the emission of CO_2_ to the atmosphere. In the case of the agricultural and industrial wastes materials, they presented minor reduction of the polluting emissions.

Other disclosure of the research was that the mixtures with SBA and MK acted as collectors of the environmental CO_2_ without provoking a decrease of the mechanical properties. This can be extremely useful for the implementation in the construction industry, helping to reduce the PC consumption and improving the disposal of waste. Additionally, the mixtures elaborated with BMA, BA, C, EP and OF also acted as CO_2_ fixers, but they did present lower mechanical resistance values. These substitutions could be applied for non-structural uses inside the construction industry, for example, acting as refurbishments or partition walls.

It is important to continue the research on potential PC substitutions from the perspective of the green economy, ecology and durability. One of the main objectives was to determine how different additions react to the carbonation process. Furthermore, these additions can proportionate other features to the concrete mixtures, which are desired for different construction processes and conditions. The materials also help to reduce the CO_2_ footprint, and because of their origins, diminish the quantity of demolitions and repairs in old buildings.

Regarding the current situation with global warming and to reduce the greenhouse emissions produced by the construction industry, it is essential to find efficient and feasible ways to diminish them. This research work demonstrates the suitability of the incorporation of waste materials from diverse industries which could be used as construction materials. The employment of these materials as substitutions helps to increase the durability of concrete structures and to passively absorb the CO_2_ of the environment.

## Figures and Tables

**Table 1:** Classification of the Portland cement substitutions.

Classification	Description	Code	(%)	Origin
Agricultural and industrial wastes	Sugarcane bagasse ash	SBA5	5	Sugar cane mill of Taretan, Michoacan, Mexico
		SBA20	20	
	Charcoal ashes	CA	5	Cuitzeo lake margins
	Brick manufacturing ash	BMA	5	Santiago Undameo, Michoacan, Mexico
	Blast-furnace slag	BFS	15	Arcelor Mittal Industry
	Bottom ash	BA	15	Arcelor Mittal Industry
	Coal	C	15	Arcelor Mittal Industry
Minerals and fibres	Expanded perlite	EP	20	Construction products with high-purity standards and quality
	Natural perlite	NP	5	
	Metakaolin	MK	20	
	Opuntia ficus-indica dehydrated fibers	OF	4	Food grade product
Fine Aggregates	Control 1, ARL and ARL2	C1	-	Lerma river margins
	Control 2, HUAJ	C2	-	Huajumbaro river margins

All the materials proposed as substitutions and the aggregates were characterized with X-Ray fluorescence spectroscopy to determine their composition and relate it with the pozzolanic activity.

**Table 2:** Results of the mechanical properties of the aggregates used.

Test	Standard	Lerma Sand	Huajumbaro Sand
Sampling	ASTM D-75-03 [66]	250 kg	250 kg
Reducing sampling	ASTM C-702 [67]	0.500 kg	0.500 kg
Bulk density (unit weight and voids)	ASTM C-29/C-29M [68]	1.353	1.226
Bulk density (unit weight)	ASTM C-29/C-29M [68]	1.444	1.331
Relative density	ASTM C-128 [69]	2–40	2.31
Specific gravity	ASTM C-128 [69]	2.39–2.48	2.24–2.36
Surface moisture (%)	ASTM C-128 [69] ASTM C-70 [70]	0.748	0.741
Absorption percentage (%)	ASTM C-128 [69] ASTM C-566 [71]	1.89	3.18
Sand equivalent value (%)	ASTM D-2419 [72]	86.97	98.25
Clay lumps and friable particles (%)	ASTM C-142 [73]	8.165	2.498

**Table 3:** EDS XRF results of the chemical composition of the materials.

	SiO_2_	TiO_2_	Al_2_O_3_	Fe_2_O_3_	MnO	MgO	CaO	Na_2_O	K_2_O	P_2_O_5_	SO_3_	PXC	Total
SBA	60.04	0.43	6.289	3.145	0.13	1.825	1.64	0.446	1.856	0.786	-	23.6	100
OF	14.58	0.83	4.287	26.77	2.423	5.995	37.456	0.032	0.024	0.814	0.8	4.79	98
CA	32.52	0.76	13.55	5.371	0.112	2.108	18.759	0.67	1.027	0.541	-	22.2	97.6
BA	27.93	0.2	6.437	2.217	0.083	1.301	49.773	0.669	1.255	0.118	3.37	5.12	95.1
BMA	19.10	0.32	8.776	2.008	0.538	4.243	27.874	0.545	6.051	1.763	0.8	27.3	98.5
BFS	36.38	0.56	10.63	0.335	0.417	10.107	37.551	0.298	0.424	0.053	1.93	0.72	96
MK	49.75	1.53	44.71	0.509	0.013	0.159	0.044	0.23	0.141	0.033	-	0.77	97.9
EP	73.59	0.13	13.43	1.166	0.045	0.197	1.333	2.918	5.013	0.02	-	1.18	99
NP	72.20	0.12	13.58	1.011	0.073	0.538	1.052	3.216	4.285	0.025	-	3.99	100
HUAJ	78.19	0.2	11.56	1.567	0.03	0.239	1.015	2.666	3.577	0.036	-	1.19	100
ARL	77.57	0.28	10.67	2.384	0.025	0.479	1.338	2.232	3.083	0.075	-	2.2	100.3
ARL2	75.25	0.32	11.92	2.689	0.026	0.568	1.482	2.219	3.124	0.077	-	2.5	100

**Table 4:** Pozzolanic activity.

Sample	SiO_2_	Al_2_O_3_	Fe_2_O_3_	Pozzolanic Activity	Acid Character
SBA	60.04	6.289	3.145	69.47	66.32
OF	14.58	4.287	26.77	45.64	19.37
CA	32.52	13.55	5.371	51.44	46.07
BA	27.93	6.437	2.217	36.58	34.41
BMA	19.1	8.776	2.008	29.88	27.87
BFS	36.38	10.63	0.335	47.35	47.01
MK	49.75	44.71	0.509	94.97	94.46
EP	73.59	13.43	1.166	88.19	87.03
NP	72.2	13.58	1.011	86.79	85.78
HUAJ	78.19	11.56	1.567	91.32	89.75
ARL	77.57	10.67	2.384	90.62	88.24
ARL2	75.25	11.92	2.689	89.86	87.16

## Data Availability

Data sharing is not applicable for this article.
